# The Sensory Equipment of Diving Lice, a Host Ecology-Based Comparative Study

**DOI:** 10.3390/insects16060574

**Published:** 2025-05-29

**Authors:** Paula Olivera, Claudio R. Lazzari, María Soledad Leonardi

**Affiliations:** 1 Instituto de Biología de Organismos Marinos, IBIOMAR-CONICET, Puerto Madryn U9120ACD, Chubut, Argentina; polivera@cenpat-conicet.gob.ar (P.O.); leonardi@cenpat-conicet.gob.ar (M.S.L.); 2 Institut de Recherche sur la Biologie de l’Insecte (IRBI), UMR7261 CNRS-University of Tours, 37200 Tours, France

**Keywords:** antennae, Echinophthiriidae, seal lice, sensilla

## Abstract

Seal lice (Anoplura) parasitize amphibious hosts, such as pinnipeds, and are uniquely adapted to an oceanic environment. As obligate, permanent ectoparasites feed on the blood of their hosts and are completely dependent on them. While studies have begun to explore general diving adaptations, research into seal lice’s sensory biology remains limited. In contrast to the vast majority of insects, including human lice, seal lice are devoid of eyes and depend on antennal sensory reception. This study aims to describe the morphology and putative function of antennal sensilla in five seal lice species: *Antarctophthirus microchir*, *A. carlinii*, *A. lobodontis*, *A. ogmorhini*, and *Lepidophthirus macrorhini*, which parasitize the South American sea lion, Weddell seal, crabeater seal, leopard seal, and southern elephant seal, respectively. The antennal structures of each species were analyzed using scanning electron microscopy, and eight morphotypes were identified: spine, cuticular lobe, sensilla squamiformia, sensilla chaetica, sensilla basiconica I and II, tuft organs, and pore organs. The morphology of sensilla and their distribution on the antennal flagellum exhibited variability among genera and species. For instance, the southern elephant louse (*Lepidophthirus macrorhini*) is characterized by the presence of sensilla squamiformia, while *Antarctophthirus* spp. are distinguished by sensilla chaetica.

## Simple Summary

Seal lice are a highly unusual group because they are permanent and obligate ectoparasites of amphibian mammals that can spend most of the year in the open ocean, diving to depths of kilometers below the surface. Although some of these insects’ adaptations to survive the extreme conditions of the deep ocean are beginning to be understood, how they sense their environment remains unexplored. Unlike human lice and most insects, seal lice have no eyes and rely only on antennae to sense their environment. Our study uses scanning electron microscopy to investigate the morphology and possible function of antennal sensilla in five seal lice species that parasitize hosts with different diving habits. Eight morphotypes of structures were identified, six of which are sensilla, with differences in external morphology and distribution in different species. The most striking difference observed across species concerns antennal mechanoreceptors. In most cases, these are thin sensilla chaetica, but in the southern elephant louse, i.e., the deepest divers, they seem to have changed and become thick and strong sensilla squamiformia.

## 1. Introduction

Seal lice (Psocodea: Echinophtiriidae) have undergone a remarkable adaptive process throughout their evolutionary history, enabling them to survive where no other insect has succeeded: the depths of the ocean. These obligate and permanent ectoparasites infest only pinnipeds and the northern river otter, keeping a close association with their hosts for their entire life cycle as hematophagous parasites. The family Echinophthiriidae comprises five genera and thirteen species: *Latagophthirus* (one species) from the North American river otter, *Proechinophthirus* (two species) from fur seals, *Echinophthirius* (one species) from true seals in the northern hemisphere, *Lepidophthirus* (two species) from monk and elephant seals, and *Antarctophthirus* (seven species) from various pinniped hosts [1,2,3].

Pinnipeds are a group of marine carnivores that includes the walrus (Odobenidae), sea lions and fur seals (Otariidae), and seals (Phocidae). A defining characteristic of this group is their extraordinary diving capacity. While otariids, e.g. South American sea lion *Otaria byronia,* usually dive to a few hundred meters, true seals double and triple these depths and can spend weeks to months at sea. Notably, adult southern elephant seal *Mirounga leonina* has been reported diving beyond 2000 m [4,5,6,7].

Like their hosts, seal lice have a terrestrial origin [8,9] and have undergone profound adaptations to survive in the marine environment. Over evolutionary time, echinophthiriids have developed distinctive morphological, behavioral, and ecological adaptations to accommodate the amphibious lifestyle of their hosts [10]. While previous research has explored various morphological adaptations—such as the role of body scales, the specialized system for closing spiracles, and the strong modifications in their legs for attachment [11,12,13,14]—few studies have focused on their sensory system. In the absence of compelling evidence indicative of an evolved visual system, it is plausible to hypothesize that these organisms predominantly depend on their antennae for the acquisition of sensory information that is indispensable for survival and reproduction.

Antennae in insects are highly specialized sensory structures that function as the primary organs for detecting mechanical, chemical, thermal, and hygric stimuli. They play a fundamental role in key behaviors such as habitat selection, foraging, mating, oviposition site selection, and both intra- and interspecific recognition. The type and distribution of antennal sensilla are influenced by factors such as sex, feeding habits, and mobility, which are determined during insect morphogenesis [15]. The typical antennae of Anoplura are usually five-segmented. In the family Echinophthiriidae, this number of segments is present in the genus *Antarctophthirus*; however, in other genera from this family, the number is reduced to four or three segments [16]. Previous studies have described antennal structures in five different seal lice species as sensilla basiconica, apical chemoreceptors, hairs, pores, and campaniform organs [16,17,18,19]. Yet, the terminology used to describe these structures remains inconsistent, and their functional significance is poorly understood.

The main aim of this study was to gain insight into the sensory structures present in five seal lice species: *Antarctophthirus microchir*, *A. carlinii*, *A. lobodontis*, *A. ogmorhini*, and *Lepidophthirus macrorhini*, which parasitize the South American sea lion, Weddell seal, crabeater seal, leopard seal, and southern elephant seal, respectively. This study qualitatively and quantitatively compared the external morphology of their antennae. We also propose a criterion for classifying sensilla based on their form and putative function. The rationale for this choice was to further investigate the adaptation of seal lice to the diving habits of their hosts, which was initiated in our laboratory several years ago [10,14,20].

## 2. Materials and Methods

### 2.1. Insects

This study focuses on adults of five species of lice. All specimens were part of the parasitological collection of the Instituto de Biología de Organismos Marinos (IBIOMAR-CONICET). *Antarctophthirus microchir* specimens were collected in Punta León, Chubut province (43°04′ S, 64°28′ W), in 2016, and *A. carlinii*, *A. ogmorhini,* and *A. lobodontis* were collected during fieldwork between 2016 and 2018, within the Antarctic Specially Protected Area (ASPA) No. 134 (64°09′ S, 60°57′ W). Finally, *Lepidophthirus macrorhini* was collected in Punta Delgada, Península Valdés, Chubut (42°46′ S, 64°38′ W), in 2017.

We analyzed 9 specimens of *A. ogmorhini*, 10 specimens of *A. lobodontis*, 7 specimens of *A. carlinii*, 10 specimens of *A. microchir*, and 12 specimens of *L. macrorhini*. For *A. ogmorhini*, all specimens were female, as this species has the least representation in the collection. For the other species, both male and female specimens were included. The specimens were cleaned with acetic acid using an entomology brush and preserved in 70% alcohol until preparation for scanning electron microscopy (SEM).

### 2.2. Scanning Electron Microscopy (SEM)

Critical Point Drying: This process was carried out using a BALZERS CPD 030 critical point dryer. Samples submerged in acetone at 10 °C were gradually replaced with liquid CO_2_ until the acetone was completely substituted. The temperature was then raised to 40 °C to evaporate the CO_2_.

SEM: The material was mounted on aluminum stubs using double-sided carbon tape and coated with a Balzers SCD 030 sputter coater, applying a gold layer of approximately 25 nm. Images were acquired using a Carl Zeiss SIGMA Field Emission Scanning Electron Microscope (FE-SEM), with a 3 keV electron beam and Zeiss Smart SEM software, employing various magnifications, and the SE2 detector (secondary electrons).

### 2.3. Sensilla Classification and Terminology

Sensory structures were located, identified, counted, and measured in diameter, length, and width as appropriate, using ImageJ software [21]. We used base width and length to classify sensilla that appeared similar but differed in shape. For measurements of each segment, we used both sides of the flagellum and pedicel, but only the dorsal side was used, as this was the only view that allowed proper observation.

The nomenclature used in this study was based on Snodgrass’s *Principles of Insect Morphology* [22] and adapted from previous studies on seal lice [17,18]. However, some structures were renamed based on the description of *Pediculus humanus* antennae by Ortega-Insaurralde et al. [23,24]. To improve the analysis and comparison among the five species, we created schematic illustrations of each antenna and the distribution of its sensillar structures in ventral and dorsal views using Adobe Illustrator 2021 [25].

## 3. Results

### 3.1. Antennae Description

All species had between 35 and 41 structures on the antennal segments. Based on their external morphology, we classified them into eight morphotypes: cuticular lobe, spines, sensilla chaetica, sensilla squamiformia, sensilla basiconica (I and II), tuft organs, and pore organs. Since there are some existing studies describing seal lice antennae [17,18], we revised the nomenclature for previously identified structures and assigned names for the first time to those that had not been described in this group until now (Table 1). Similarly, based on their external morphology, we proposed a functional classification for each structure.

**Figure 1:**
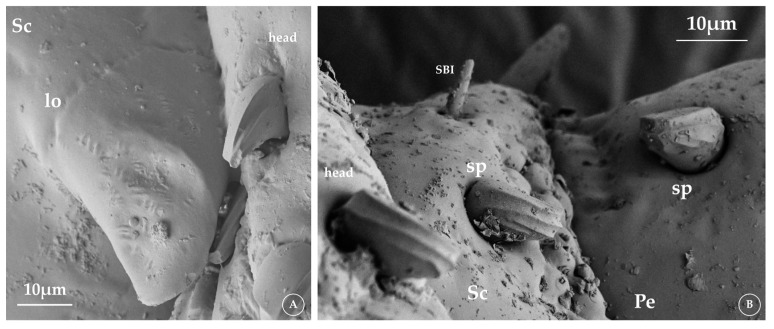
Detail of structures located on the scape and pedicel. (**A**) *Lepidophthirus macrorhini* ventral side, cuticular lobe (lo). (**B**) *Antarctophthirus ogmorhini* dorsal side, showing spines (sp) on scape (Sc) and pedicel (Pe). Abbreviations: SBI—sensilla basiconica I.

There is a similar structure to the cuticular lobe (Figure 1A) described by Miller Jr. [17] in other species. It is a cone-like cuticular projection located on the ventral side of the scape base in *Lepidophthirus macrorhini* and lacks pores on its surface. The genus *Antarctophthirus* exhibits spines (Figure 1B) on the dorsal side of the scape and pedicel. These spines are short, possess a well-developed socket, and have a rough surface. These structures were previously described as campaniform organs in *Echinophthirius horridus*, *Proechinophthirius fluctus*, and *A. callorhini* [17]. However, after a detailed analysis of their shape, we suggest that they are the same setae found on the head and differ from the campaniform organs described in the literature [22]. Although not sensory, the morphological features of both structures in the louse antennae may aid in species identification and ultimately play a functional role in the interaction of stimuli with sensilla underwater.

### 3.2. Sensilla Type and Its Morphology

**Figure 2:**
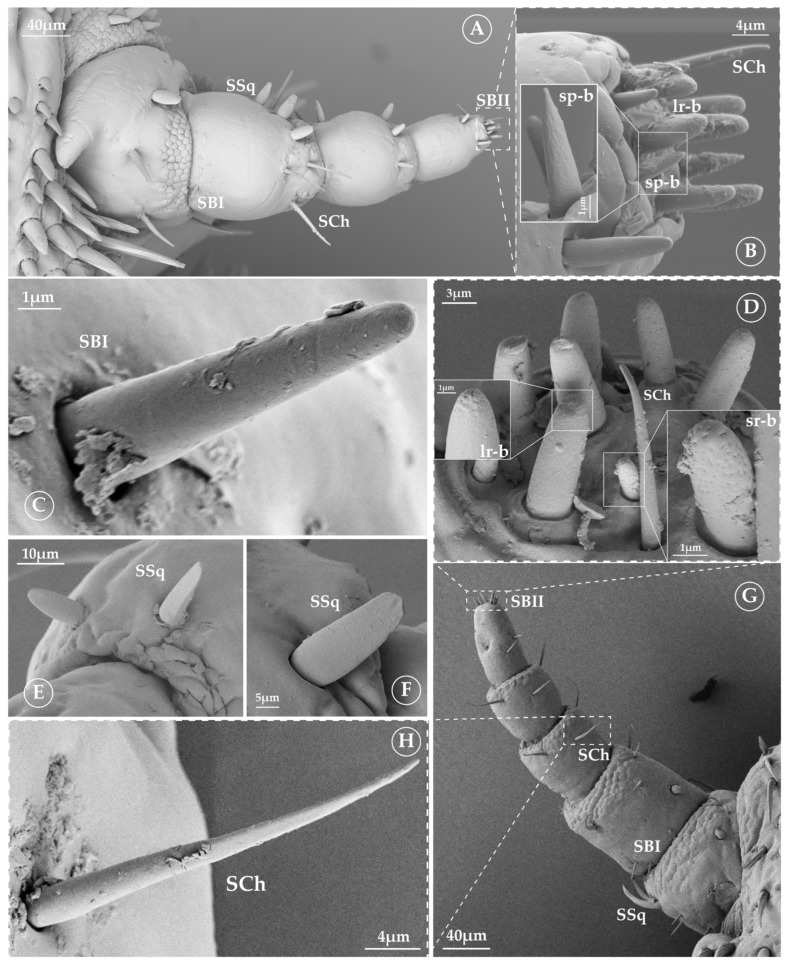
Sensilla morphotypes. (**A**) *Lepidophthirus macrorhini* antenna (ventral view), showing sensilla distribution. (**B**) Detail of flagellomere 2 (*L. macrorhini*) with sensilla basicanica II (SBII), pores can be observed on the short pointed basiconica (sp-b). (**C**) Sensilla basiconica I (SBI). (**D**) Detail of flagellomere 3 (*Antarctophthirus microchir*); each SBII is magnified—large rounded basiconica (lr-b) and short rounded basiconica (sr-b)—showing porous surfaces. (**E**) Sensilla squamiformia (SSq) from *L. macrorhini*. (**F**) Sensilla squamiformia from *Antarctophthirus* spp. (**G**) *A. microchir* antenna (dorsal view), showing sensilla distribution. (**H**) Detail of sensilla chaetica (SCh).

Six types of possible sensory structures can be recognized to be present in the antennae of seal lice. They are shown in Figure 2; Table 1 summarizes some of their characteristics and putative function, and Table 2 their dimensions. Below is a brief description of each type and how they occur in the different genera.

#### 3.2.1. Sensilla Squamiformia (SSq)

In both genera analyzed, we found this type of sensilla in almost all segments of *Lepidophthirus* and, at least, in the first segment of *Antarctophthirus*. SSq are scale like, with a wide base and a flattened appearance. They may have either a short or long process (Figure 2E,F). Although in one genus they exhibit a structure longer than in the other, the width at the base is similar.

#### 3.2.2. Sensilla Chaetica (SCh)

**Figure 3:**
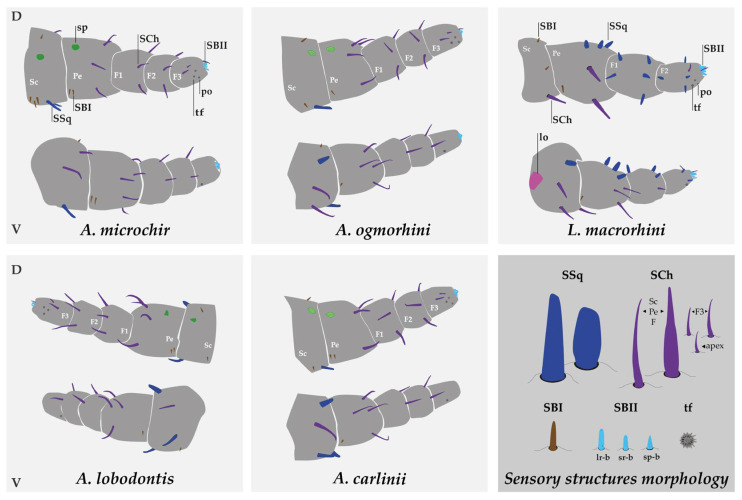
Scheme of sensilla distribution across species and sensilla morphology. The dorsal and ventral views display all sensory structures. In each quadrant, the dorsal side is shown above the ventral side. The bottom right quadrant (darker background) illustrates the morphology of the sensilla described in this study, with sizes shown in relative proportion. For SSq and SCh, both morphotypes are represented according to genus. The one on the left corresponds to *Antarctophthirus* and the one on the right to *Lepidophthirus*. Abbreviations: sp—spine (green); lo—cuticular lobe (pink); SCh—sensilla chaetica (violet); SSq—sensilla squamiformia (blue); SBI—sensilla basiconica I (brown); SBII—sensilla basiconica II (light blue and orange); tf—tuft organ (gray); po—pore organ (gray); Sc—scape; Pe—pedicel; F1–F3—flagellomeres 1–3.

Sensilla chaetica are typical spine-like structures, inserted into a socket and slightly flattened at their distal half (Figure 2H). They are present in all antennal segments of both genera. Sensilla chaetica exhibit slight morphological differences between genera. In *Antarctophthirus*, they appear predominantly cylindrical, maintaining a relatively uniform thickness along their length. In *Lepidophthirus*, they may have very subtle lateral expansions that could resemble small flanges or wing-like projections, though these features are not always clearly distinguishable (Figure 3).

#### 3.2.3. Sensilla Basiconica (SB)

SB can be divided into two subtypes: SBI and SBII (Figure 2B–D and Figure 3), depending on the presence or absence of pores. SBI is a minute peg found on the ventral and dorsal sides of the scape and pedicel; it lacks pores on its surface and functions as a mechanoreceptor. Meanwhile, SBII can be either a minute peg or a cone; it is located at the apex of the last flagellomere, with visible pores and a possible chemoreceptive function.

In the last flagellomere, we identified nine to ten SBII (Figure 2B,D) that we classified in three morphological subtypes (Figure 3): large rounded basiconica (lr-b), short rounded basiconica (sr-b) and short point-like basiconica (sp-b). The lr-b (Figure 2B) exhibited multiple pores in the extreme distal and a proximal unique pore. The sr-b and sp-b (Figure 2B,D) were characterized by the presence of pores all along their surface.

#### 3.2.4. Tuft Organ (tf)

**Figure 4:**
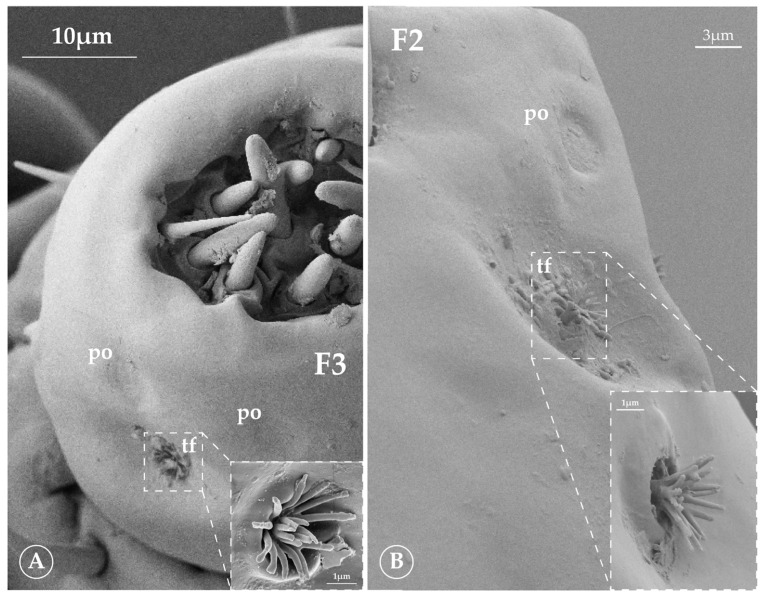
Final flagellomere; detail of the tuft organ on both genera. (**A**) *Antarctophthirus*. (**B**) *Lepidophthirus*. Abbreviations: po—pore organ; tf—tuft organ.

The sensilla referred to as the tuft organ (Figure 3 and Figure 4) were first described by Wigglesworth [26] in *Pediculus humanus*. However, they are also present in this family of lice. This structure consists of a deep, circular pit from which a first row of pegs emerges, with a second row of pegs inside. This arrangement is observed in *Antarctophthirus*. In contrast, *Lepidophthirus macrorhini* appears to have a single row of pegs that bifurcates distally to form a small V-like shape.

#### 3.2.5. Pore Organ (po)

Finally, pore organs (Figure 4) appear as depressions located on the final antennal segment, adjacent to the distal tuft organ, with pores or grooves radiating from the center [23]. These structures resemble those described by Miller [17] for three species of seal lice from the northern hemisphere.

### 3.3. Sensory Structure Description and Distribution for Species

**Figure 5:**
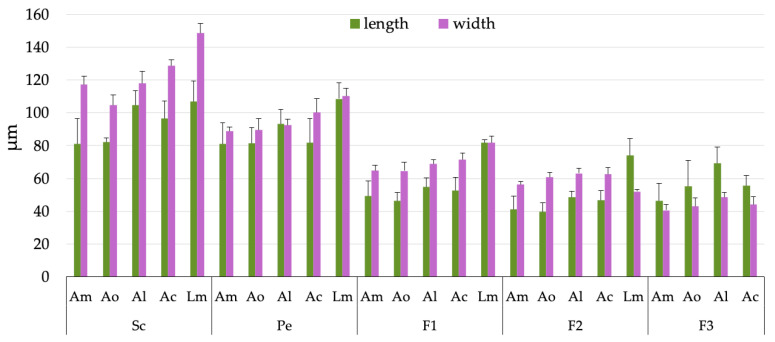
Dimensions (in micrometers) of all segments in five species of seal lice. Green bars represent length, and purple bars represent width, both with their standard deviations (SDs). Abbreviations: *Am*—*Antarctophthirus microchir*; *Ao*—*A. ogmorhini*; *Al*—*A. lobodontis*; *Ac*—*A. carlinii*; *Lm*—*Lepidophthirus macrorhini*; Sc—scape; Pe—pedicel; F1, F2, F3—flagellomeres 1, 2, and 3, respectively.

The distribution of these structures is similar in all species. The scape bears between six and eight sensilla squamiformia and/or chaetica, the pedicel between nine and twelve, and the flagellum between twenty and twenty-three. The pedicel has the highest number of mechanoreceptors, while flagellomere 3 (F3) contains the chemoreceptors (Figure 3, Table 1). In addition, F3 has two tuft organs and two pore organs, the arrangement of which varies between species. These structures may have thermo- and hygroreceptive functions. In *Antarctophthirus*, one tuft organ is located at the base on the ventral side, while the other is positioned near the two sensilla chaetica on the laterodorsal side. In contrast, *Lepidophthirus* has both tuft organs next to each other on the lateral side, near the apex of the flagellomere. The two pore organs appear to be anatomically associated with the distal tuft organs [17]. In general, the width of the sensilla chaetica on the dorsal side is slightly greater than that on the ventral side of the pedicel, F1, and F2 in both genera. The mean of the individual sensory structures is shown in Table 2. In addition, the mean of segment length and width are shown in Figure 5.

#### 3.3.1. Description of *Antarctophthirus microchir*

The scape is approximately 1.45 times wider than long and exhibits three types of sensilla. On its dorsal side, there are four SBI, while the ventral side has two SCh and the lateral side has two SSq. A spine is present on the distal end of the dorsal side.

The pedicel is about 1.10 times wider than long. It bears five SCh on the ventral side and three dorsal, also there are three or four SBI (two dorsal, one or two ventral). A spine, similar to that of the scape, is located proximally on the dorsal side, near the first spine.

The flagellum consists of three segments (hereafter referred to as F1, F2, and F3). F1 and F2 are around 1.30 and 1.37 times wider than long, respectively, whereas F3 is the only segment that reverses this trend, about 1.14 times longer than wide. F1 and F2 have a similar sensilla distribution, each bearing two SCh on the dorsal side and two on the ventral side. On F1, one of the dorsal SCh is slightly larger than the other three. F3 has two dorsal SCh that are slightly shorter than those on F1 and F2. Additionally, F3 exhibits two tuft organs and two pore organs adjacent to the distal tuft organ. Each tuft organ consists of a deep, circular pit from which sixteen pegs emerge in a first row, with a second row of four pegs inside. At the apex, there are one Sch and two types of SBII: six large rounded basiconica (lr-b) and four short rounded basiconica (sr-b). In what follows, the structures are conserved among the species of the genus, remaining as described in *A. microchir* unless otherwise stated.

#### 3.3.2. Description of *A. ogmorhini*

The scape is 1.27 times wider than long. On its dorsal side, there are two SBI and one SSq, while the ventral side has two SCh and one SSq. A spine is present on the distal end of the dorsal side.

The pedicel is 1.10 times wider than long. On its dorsal side, it bears four SCh and one SBI, while the ventral side has three SCh and one SBI. A spine, similar to that of the scape, is located proximally on the dorsal side, near the first spine.

The first two segments of the flagellum are 1.40 and 1.53 times wider than long, while the third is 1.28 times longer than wide. F1 and F2 have the sensilla distribution from the genera, with two SCh on the dorsal side and two on the ventral side. On F2, one of the dorsal SCh is slightly larger than the other three. F3 has two dorsal SCh, two tuft organs, and two pore organs adjacent to the distal tuft organ. Each tuft organ consists of a deep, circular pit from which fourteen pegs emerge in a first row, with a second row of three pegs inside. At the apex, there is one SCh and two types of basiconica II: six lr-b and three sr-b.

#### 3.3.3. Description of *A. lobodontis*

The scape is 1.13 times wider than long. On its dorsal side, there are two SBI and one SSq, while the ventral side has one SCh, one SBI, and two SSq. A spine is present on the distal end of the dorsal side.

The pedicel has equal length and width, with a ratio of 1.00. On its dorsal side, it bears three SCh and two SBI, while the ventral side has four SCh and one SBI. A spine, similar to that of the scape, is located proximally on the dorsal side, near the first spine.

The flagellum consists of three segments. As in other species of *Antarctophthirus*, the first two segments are broader than they are long, with a ratio of about 1.25 and 1.30, respectively, while the third is 1.42 times longer than wide. Its sensilla distribution is characteristic of the genus. On F1 and F2, one SCh is slightly larger than the other three. F3 exhibits two tuft organs and two pore organs adjacent to the distal tuft organ. Each tuft organ consists of a deep, circular pit from which twelve pegs emerge in a first row, with a second row of four pegs inside. At the apex, there is one SCh and two types of SBII: six lr-b and four sr-b.

#### 3.3.4. Description of *A. carlinii*

The scape is 1.33 times wider than long. On its dorsal side, there are two SBI and one SSq, while the ventral side has one SCh and two SSq. A spine is present on the distal end of the dorsal side.

The pedicel is 1.23 times wider than long. On its dorsal side, it bears two SCh and two SBI, while the ventral side has four SCh and one SBI. A spine is located proximally on the dorsal side, near the one in the scape.

The flagella F1 and F2 are 1.36 and 1.34 times wider than long, respectively, while the third is 1.26 times longer than wide. Their sensilla distribution is characteristic of the genus. On F1 and F2, one SCh is slightly larger than the other three. F3 exhibits two tuft organs and two pore organs adjacent to the distal dorsolateral tuft organ. The other one is on the proximal ventral side. Each tuft organ consists of a deep, circular pit from which thirteen pegs emerge in a first row, with a second row of six pegs inside. At the apex, there is one SCh and two types of SBII: six lr-b and three sr-b.

#### 3.3.5. Description of *Lepidophthirus macrorhini*

The antennae of *Lepidophthirus* consist of four segments: the scape, pedicel, and a two-segmented flagellum. The sensory structures of *L. macrorhini* are predominantly represented by SSq. The SSqII are shorter than those from *Antarctophthirus*. Despite having only two flagellomeres, they present a similar number of sensilla to the species from the genera *Antarctophthirus*. Regarding the shape of the SBII, this species has a short pointed basiconica (sp-b) type, instead of a short rounded (sr-b) type presented in *Antarctophthirus*.

In the specimen analyzed, the scape was clearly broader than long, with a width-to-length ratio of approximately 1.39. The pedicel and the first flagellomere were nearly as long as they were wide, with length-to-width ratios of about 0.99 and 1.00, respectively, indicating a roughly quadrate shape. In contrast, the second flagellomere (F2) was distinctly longer than wide, with a length-to-width ratio of approximately 1.42.

The scape is 1.39 times broader than long. On its dorsal side, there are two SBI and one SCh, while the ventral side has two SCh and one SSq, and on the proximal side, there is a cuticular lobe.

The pedicel is nearly as long as is wide, with a ratio of about 1.01. On its dorsal side, it bears one SBI, two SCh, and two SSq, while the ventral side has three SCh, one SBI, and one SSq. The width of the SCh on the dorsal side is slightly greater than that of those on the ventral side in the pedicel.

The flagellum F1 is approximately as wide as it is long, with a ratio of about 1.00, while F2 is distinctly longer than wide, with a length-to-width ratio of approximately 1.42. F1 bears five SSq on the dorsal side, while the ventral side has one SBI and only one SSq. F2 has one SCh and three SSq on the dorsal side and two SSq on the ventral side. Additionally, two tuft organs are present lateroventrally at the distal part, positioned next to each other, with adjacent pore organs. Each tuft organ consists of a deep, circular pit from which a stalk emerges, branching into ten principal pegs, at least six of which branch at their mid-distal point to form twelve smaller pegs. At the apex, there is one SCh and two types of SBII: six lr-b and four sp-b.

## 4. Discussion

In Phthiraptera, the antennae typically consist of five segments: the scape, the pedicel, and three flagellomeres [16]. This arrangement is observed in species of the genus *Antarctophthirus*, whereas *Lepidophthirus* displays a modified structure with a two-segmented flagellum. Based on this distinction, we examined and quantified interspecific differences in the morphology, distribution, and number of sensory structures across the five lice species. In all investigated seal louse, the antennal segments are generally wider than they are long, except for the last flagellomere (F2 in *Lepidophthirus* and F3 in *Antarctophthirus*), which is longer than wide.

Despite the reduction in the number of flagellomeres in *Lepidophthirus*, both genera exhibited a similar number of sensory structures. In total, between 35 and 41 different structures are present in all species studied, which can be classified into eight morphotypes: non-sensory spines and cuticular lobes and six types of putative sensory structures. Based on the external morphology, we identified sensilla squamiformia (SSq), sensilla chaetica (SCh), sensilla basiconica I and II (SBI and SBII), tuft organs (tf), and pore organs (po).

In *Antarctophthirus*, spines were located on the dorsal side of the scape and pedicel and appeared similar to those found on the head, as their shape varied across the four species of the genus. These spines may subtly differ in size and surface roughness. Meanwhile, the cuticular lobe was present only in *L. macrorhini*, on the ventral side of the proximal scape.

Regarding the predominant sensory structures, *Antarctophthirus* is characterized by a high prevalence of SCh across the antennae, whereas in *Lepidophthirus,* SSq is more abundant. The length of SCh remained consistent across all segments, except for the final segment, where it was reduced by half. In contrast, SSq in *Lepidophthirus* gradually decreased in size along the antenna. In general, sensilla on the dorsal side were wider and longer than those on the ventral side.

Among *Antarctophthirus* species, SSq in the scape showed similar length values, as did SBI. However, SCh exhibited greater variation, with *A. carlinii* displaying the highest value. The pedicel was characterized by SCh, which had similar mean lengths across the species, and SBI, which showed only minor differences in length. Additionally, notable differences were observed in the length of SCh on the flagellum. In *A. microchir,* one SCh was longer than the others on F1, while in *A. ogmorhini*, this was observed in F2. Meanwhile, *A. lobodontis* and *A. carlinii* had one SCh longer than the others in both F1 and F2. In contrast, *L. macrorhini* had the lowest mean sensilla length value (compared to *Antarctophthirus* spp.), except for SCh in the pedicel.

The evaluation of SBII, tuft organs, and pore organs was particularly challenging due to the particular structure of these organs and the technical challenge associated with collecting large numbers of individuals in the field and properly cleaning the antennae. Many of these structures were either broken or covered by grime, complicating the analysis. However, we were able to identify and count tuft organ and their components. Pore organs, on the other hand, were only visible in a few specimens.

Although early taxonomic descriptions of seal lice included general morphological features, neither those descriptions nor later redescriptions have addressed sensory structures in depth [2]. In this study, we reviewed the nomenclature used for these structures in the group and found no general consensus. Thus, we proposed a standardized nomenclature based on the classical categorization by Snodgrass’s *Principles of Insect Morphology* [22], as well as previous studies on *Echinophthirius horridus*, *Proechinophthirius fluctus*, *Antarctophhirus callorhini* [17], and *P. zumpti* [18], along with studies on *Pediculus humanus* [23,24].

Previous studies by Castro et al. [18] also identified setae on the scape that resemble spines found on the head or, as suggested by Miller [17], may correspond to campaniform organs. This suggests that such structures could be a characteristic feature of the group. Additionally, Miller identified tuft organs in three species and suggested that the tuft organs in Anoplura should be classified as sensilla basiconica. He may have also recognized pore organs and ventral lobes. However, in *P. zumpti*, the authors referred to tuft organs as “placodeal olfactory chemoreceptors”. Since the terminology used for sensory structures often depends on the preference of the authors, we propose adopting the term “tuft organ” to establish a unified criterion—at least for this group—given that this is the first study to comprehensively describe the antennae of five out of the thirteen known species.

In general, nonporous sensilla inserted in a flexible socket are assumed to function as mechanoreceptors rather than chemoreceptors [22]. Based on this, we assigned a potential mechanosensory function to sensilla squamiformia, chaetica, and basiconica I. The apex of the last flagellomere exhibits sensilla basiconica II, which have pores on their surface—a characteristic feature of chemoreceptive sensilla. Studies on human lice suggest that these structures are involved in the gustatory and olfactory systems [23,24]. The tuft organ was previously described by Miller as a humidity receptor, and Insaurralde et al. [23] further proposed its role as a thermoreceptor in *Pediculus humanus*. Additionally, they identified the pore organ as a chemo-thermosensory structure [23].

This study contributes to the current knowledge of seal lice and represents a first step toward characterizing the sensory structures of this group. These findings open the possibility of conducting phylogenetic analyses based on sensillar characteristics. The characterization of antennal structures has significant taxonomic value and is also relevant to evolutionary biology and the mechanisms of olfactory recognition involved in behavioral control [17,27]. Future studies, including the study of electrophysiological responses of antennal sensilla, as well as molecular analyses, could shed light on whether variations at the level of the peripheral nervous system contribute to sensory adaptation in this particular group of insects.

## Figures and Tables

**Table 1:** Classification and distribution of antennal structures. For each species, the position of each structure—scape (Sc), pedicel (Pe), and flagellomeres 1–3 (F1–F3)—is indicated, followed by the number (#) observed in parentheses. The putative function of each structure is also provided. The corresponding nomenclature from previous studies is shown in the final columns. Abbreviations: *Am*—*Antarctophthirus microchir*; *Ac*—*A. carlinii*; *Al*—*A. lobodontis*; *Ao*—*A. ogmorhini*; *Lm*—*Lepidophthirus macrorhini*; *Eh*—*Echinophthirius horridus*; *Pf*—*Proechinophthirus fluctus*; *Acal*—*A. callorhini*; *Pz*—*P. zumpti*; SSq—sensilla squamiformia; SCh—sensilla chaetica; SBI—sensilla basiconica I; SBII—sensilla basiconica II; lr-b—large rounded basiconica; sr-b/sp-b—short rounded basiconica/short pointed basiconica; tf—tuft organ; po—pore organ.

								Correlative Nomenclature	
			*Lm*	*Al*	*Ao*	*Ac*	*Am*	*Eh/Acal/Pf*	*Pz*
putative function	structure		position (#)	position (#)	position (#)	position (#)	position (#)	Miller Jr., 1971 [17]	Castro et al., 2002 [18]
mechanosensory	SSq		Sc(1), Pe(3), F1(6), F2(5)	Sc(3)	Sc(2)	Sc(3)	Sc(2)	undescribed	undescribed
mechanosensory	SCh		Sc(3), Pe(5), F1(1), F2(2)	Sc(1), Pe(7), F1(4), F2(4), F3(3)	Sc(2), Pe(7), F1(4), F2(4), F3(3)	Sc(1), Pe(6), F1(4), F2(4), F3(3)	Sc(2), Pe(8), F1(4), F2(4), F3(3)	hair	caeloconic senosira tactile
mechanosensory	SBI		Sc(2), Pe(2)	Sc(3), Pe(3)	Sc(2), Pe(2)	Sc(2), Pe(3)	Sc(4), Pe(4)	undescribed	undescribed
chemosensory (olfactory)	SBII	lr-b	F2(6)	F3(6)	F3(6)	F3(6)	F3(6)	undescribed	apical chemoreceptors
chemosensory (olfactory)		sr-b/sp-b	F2(4)	F3(4)	F3(3)	F3(3)	F3(4)	undescribed	
thermo-hygro sensory	tf		F2(2)	F3(2)	F3(2)	F3(2)	F3(2)	sensilla basiconica	placodeas olfactory chemoreceptor
chemo-thermo sensory	po		F2(2)	F3(2)	F3(2)	F3(2)	F3(2)	pore organ	undescribed
-	spine		-	Sc(1), Pe(1)	Sc(1), Pe(1)	Sc(1), Pe(1)	Sc(1), Pe(1)	campaniform organ	spines
-	cuticular lobe		Sc(1)	-	-	-	-	lobe	undescribed

**Table 2:** Sensilla dimensions. Mean length (in µm) and standard deviation are provided for each sensillum type by antennal segment and species. Values marked as “unknown” indicate measurements were not available. Abbreviations: Sc—scape; Pe—pedicel; F1–F3—flagellomeres 1–3; *Lm*—*Lepidophthirus macrorhini*; *Ac*—*Antarctophthirus carlinii*; *Al*—*A. lobodontis*; *Ao*—*A. ogmorhini*; *Am*—*A. microchir*.

			*Am*	*Ao*	*Al*	*Ac*	*Lm*
			length ± SD	length ± SD	length ± SD	length ± SD	length ± SD
**Sc**	Squamiformia		33.28 ± 4.97	30.43 ± 6.54	31.65 ± 5.61	34.57 ± 5.24	23.4 ± 1.54
	Chaetica		26.39 ± 7.78	35.72 ± 8.65	37.96 ± 5.00	50.01 ± 7.35	36.77 ± 6.07
	Basiconica I		11.31 ± 2.73	9.32 ± 0.96	12.73 ± 2.68	10.76 ± 1.90	11.41 ± 1.47
**Pe**	Squamiformia		-	-	-	-	21.4 ± 3.40
	Chaetica		27.61 ± 7.26	28.36 ± 7.71	29.73 ± 8.01	30.48 ± 7.21	44.01 ± 10.38
	Basiconica I		11.06 ± 2.31	8.25 ± 1.22	14.59 ± 7.16	8.90 ± 2.32	10.11 ± 1.44
**F1**	Squamiformia		-	-	-	-	18.99 ± 4.98
	Chaetica		26.94 ± 7.97	27.7 ± 7.33	27.28 ± 10.35	28.27 ± 7.83	19.29 ± 4.90
**F2**	Squamiformia		-	-	-	-	11.34 ± 2.23
	Chaetica		24.8 ± 7.56	27.48 ± 6.15	29.29 ± 12.57	22.94 ± 5.59	15.05 ± 2.08
**F2/F3**	Squamiformia		-	-	-	-	-
	Chaetica		12.09 ± 4.44	11.92 ± 6.8	14.88 ± 8.19	11.27 ± 3.4	
	Basiconica II	lr-b	9.16 ± 1.87	7.23 ± 1.30	8.68 ± 1.07	unkown	8.40 ± 1.26
		sr-b	6.01 ± 1.14	5.27 ± 1.11	5.58 ± 0.41	unkown	-
		sp-b	-	-	-	-	6.58 ± 0.96
	Pore Organ		4.76 ± 0.46	unkown	unkown	3.80 ± 0.27	4.35 ± 0.22
	Tuft Organ		4.51 ± 0.67	3.76 ± 0.80	4.60 ± 0.41	unkown	3.53 ± 0.49

## Data Availability

The original contributions presented in this study are included in the article. Further inquiries can be directed to the corresponding author.

## Abbreviations

The following abbreviations are used in this manuscript:

SCh	Sensilla chaetica
SSq	Sensilla squamiformia
SB	Sensilla basiconica
sp-b	Short pointed basiconica
lr-b	Large rounded basiconica
sr-b	Short rounded basiconica
*Am*	*Antarctophthirus microchir*
*Ac*	*Antarctophthirus carlinii*
*Al*	*Antarctophthirus lobodontis*
*Ao*	*Antarctophthirus ogmorhini*
*Lm*	*Lepidophthirus macrorhini*
Sc	Scape
Pe	Pedicel
F	Flagellomere
